# Women’s empowerment for abortion and family planning decision making among marginalized women in Nepal: a mixed method study

**DOI:** 10.1186/s12978-021-01087-x

**Published:** 2021-02-04

**Authors:** Heera KC, Mangala Shrestha, Nirmala Pokharel, Surya Raj Niraula, Prajjwal Pyakurel, Surya Bahadur Parajuli

**Affiliations:** 1 Department of Nursing, Birat Health College, Purbanchal University, Biratnagar, Nepal; 2 Department of Maternal Health Nursing, B. P. Koirala Institute of Health Sciences, Dharan, Nepal; 3 School of Public Health and Community Medicine, B. P. Koirala Institute of Health Sciences, Dharan, Nepal; 4 Department of Community Medicine, Birat Medical College Teaching Hospital, Tankisinuwari, Morang, Nepal

**Keywords:** Women’s empowerment, Marginalized, Abortion, Family planning, Decision-making, Reproductive health, Nepal

## Abstract

**Background:**

Women’s empowerment is multidimensional. Women’s education, employment, income, reproductive healthcare decision making, household level decision making and social status are vital for women’s empowerment. Nepal is committed to achieving women empowerment and gender equality, which directly affects the reproductive health issues. This can be achieved by addressing the issues of the poor and marginalized communities. In this context, we aimed to find the association of women’s empowerment with abortion and family planning decision making among marginalized women in Nepal.

**Methods:**

A cross sectional study was conducted at selected municipalities of Morang district of Nepal from February 2017 to March 2018. A mixed method approach was used, where 316 married marginalized women of reproductive age (15–49 years) and 15 key informant interviews from representative healthcare providers and local leaders were taken. From key informants, data were analysed using the thematic framework method. Findings obtained from two separate analyses were drawn together and meta inferences were made.

**Results:**

Women’s empowerment was above average, at 50.6%. Current use of modern contraceptives were more among below average empowerment groups (p 0.041, OR 0.593 C.I. 0.36–0.98). We could not find any statistically significant differences among levels of women’s empowerment, including those women with abortion knowledge (p 0.549); family planning knowledge (p 0.495) and women’s decision for future use of modern contraceptives (p 0.977). Most key informants reported that unsafe abortion was practiced.

**Conclusions:**

Women’s empowerment has no direct role for family planning and abortion decision making at marginalized communities of Morang district of Nepal. However, different governmental and non-governmental organizations influence woman for seeking health care services and family planning in rural community of Nepal irrespective of empowerment status.

## Plain English summary

Sustainable Development Goal (SDG-5) addresses women and girls with equal access to education, healthcare, decent work, and their representation in political and economic decision-making processes that fuel sustainable economies and benefit societies and humanity. Women's empowerment, abortion and family planning are inter related. Easy access to contraceptive devices help reduce unsafe abortion, unintended pregnancy, reduce maternal and child health morbidity and mortality. “Nepal is committed to achieving women’s empowerment and gender equality in line with SDG-5”. To achieve this, there needs to be equitable involvement of all women in reproductive health service. Hence, we conducted our study among marginalized married women of reproductive age group in a district of Nepal. Our objective was to find the association of women’s empowerment with abortion and family planning decision making. First, we collected data on women’s empowerment measures, knowledge and practices on abortion and family planning methods from the marginalized women and concurrently from the same locality, we conducted key informant interviews to explore further. We found that women’s empowerment has no significant association on abortion and family planning decision making among marginalized women. Illegal practices of abortion was reported from key informants. Current use of contraceptives devices were more in women who were below average women’s empowerment than above average women’s empowerment. Our result concluded that availability and easy access to healthcare facilities, easy transportation and support from different governmental and non-governmental organisations can influence the abortion and family planning decisions even if women are empowered or not.

## Background

Women's empowerment is determined by women's sense of self-worth, their right to determine choices; right to have access to opportunities and resources; right to have power to control their own lives within and outside the home; and their ability to influence the direction of social change [1, 2]. Nepal became the signatory of International Conference on Population and Development (ICPD) in Cairo, Egypt in 1994, which emphasized women’s empowerment and reproductive rights issues. Since then, it has been a key part in Millennium Development Goal 3 (MDG) and now on SDG-5 [3]. SDG 5 addresses women and girls with equal access to education, healthcare, decent work, and their representation in political and economic decision-making processes that fuel sustainable economies and benefit societies and humanity [4]. Nepal Demographic and Health Survey (NDHS) measures women’s empowerment in terms of employment, earnings, control over earnings, and magnitude of earnings relative to those of partners, participation in household decision-making and attitudes towards intimate partner violence (IPV) [5]. According to 2011 Nepal census, Dalit constitutes 13.6 percent of the total population [6]. Dalit by virtue of caste based discrimination and untouchability, are most stigmatized in social, economic, educational, political and religious fields, and are deprived of human dignity and social justice [7]. Dalit women need to be included in order to achieve the country's commitments to global family planning goals and to reach a modern contraceptive prevalence rate of 52% by 2020, the target set by the Nepal National Health Sector Strategy 2016–2021 [8]. Evidence shows that improvement in the welfare of women and closing inequality gaps can improve maternal and child health, reduce mortality and contribute to socio economic development [9]. Nepal is committed to achieving women empowerment and gender equality as per SDG 5 of the United Nation [10]. This goal can be achieved by addressing the issues of the poor and marginalized community. Women's empowerment, abortion and family planning are inter related. Empowering women help exercise free choices, right to control fertility, right to take an autonomous decision on healthcare seeking behaviour, mobility, reproductive rights, ownership of assets, participation in social group and increase awareness [11, 12]. However, there is a significant barrier on knowledge on abortion laws and access to safe abortion practices. Safe abortion practices and family planning methods are powerful tools to monitor women’s status within the community. Easy access to contraceptive devices help reduce unsafe abortion, unintended pregnancy, reduce maternal and child health morbidity and mortality. Women would be able to take full advantage of the broader life opportunities as they move beyond their roles of wives and mothers [11]. In order to have this type of decision-making power, women’s empowerment is an essential precondition [11, 13]. Factors of woman’s empowerment contributing to abortion and family planning decision making is of paramount importance to reduce morbidity and mortality related to unsafe abortion and to increase access to family planning services. In this context, we aimed to find the association of women’s empowerment with abortion and family planning decision making among marginalized women in Nepal.

## Methods

A cross sectional study was conducted at selected municipalities of Morang district of Nepal from February 2017 to March 2018. A concurrent mixed method (QUAN + qual) approach was used, where 316 married marginalized women of reproductive age (15–49 years) and 15 key informant interviews from representative healthcare providers and local leaders were taken. Multistage sampling method was used to select participants from Morang district, which has 17 municipalities. In first stage, 18 marginalized communities from 11 municipalities were included based on recommendation of the District Public Health Office (DPHO), Morang, Nepal. We operationalized marginalized women as those women who are socially, economically, politically and by ethnicity deprived. The marginalized females were from Dalit, Janajati, Muslim and Madhesi ethnicities of Nepal. In the second stage, households were selected. First household was selected from one corner of the community and then each alternate household were selected. If the participants were not available, then an adjacent household was taken. In the third stage, one participant from a selected household available at the time of data collection, meeting the eligibility criteria was taken. In case of more than one eligible participant in a household, the youngest eligible participant was selected. For key informants, representative key persons such as local leaders, female community health volunteers (FCHV), health post in charges, primary health center auxiliary nurse midwives were selected purposively. The total time taken for each key informant interview was 25–30 min. The interview conduct and reporting adheres to the Consolidated Criteria for Reporting Qualitative Research (COREQ) [14]. The purpose of the research was explained to each participants and then informed written consent was obtained. The interview of each participant was taken in a private room. Participants were informed that they have the right to withdraw from participation at any time of interview.

The independent variable was women’s empowerment. Generally, proxy indicators are commonly used to measure empowerment due to its multifaceted nature [15]. As there is not a standard tool to measure women’s empowerment, consensus was made to measure women’s empowerment in terms of women's biodemographic and reproductive health measures. Women’s empowerment score was calculated based on 15 different variables which includes education, occupation, socioeconomic status, age at marriage, child mortality of participant, intended pregnancy, desire for future pregnancy, sex preference of child, ideal number of children, contraception use decision making, able to refuse sexual intercourse, ask husband to use condom during intercourse, general healthcare decision making, decision over use of income and involvement in any social group (mothers group/saving group/women group). Maximum score for each variable was given 1 and the possible highest score was 15. The median score of all the variables of women’s empowerment was calculated to be 7.5. Accordingly, it was categorised into two groups after statistical consultations. Women’s empowerment was considered above average if the score was more than or equal to 7.5 (median value) and below average if it was below the median value. Dependent variables were abortion knowledge, abortion practice, family planning knowledge and family planning practice. The two variables (knowledge on fertile period and knowledge on abortion law: scored 1 for each right response) were used to calculate abortion knowledge where median value for abortion knowledge was 1. Above or equal to median value was considered as having abortion knowledge and below it was considered having no abortion knowledge. The single variable was used to assess the abortion practice. It was scored 1 for good practice and 0 if not. The family planning knowledge was calculated asking participants about their knowledge on modern contraceptives methods: female sterilisation, male sterilisation, intrauterine contraceptive devices (IUCD), Depo-Provera, implant, oral contraceptive pills (OCPs) and male condom. Participants who heard about each method was marked as yes and scored 1. The maximum knowledge score was 7 and minimum was 0. We operationalized the definition of adequate and inadequate knowledge in our context based on median cut off score. Henceforth, we calculated median score for family planning knowledge which was 6. Participants scoring above and equal to median value were considered having adequate knowledge of family planning and below having inadequate knowledge. The family planning practices were assessed by two variables: currently practicing any modern contraceptive methods and thinking of using contraception in the near future. Participants responding “yes” to each variable was scored 1 and “no” was scored 0.

### Data analysis

#### Quantitative

We performed univariate analysis to find the sociodemographic characteristics. For bivariate analysis, Chi square test was used to find the association between dependent and independent variables. Odds ratio and confidence interval were calculated and p value less than 0.05 at 95% confidence interval was considered as statistically significant.

### Key informant interviews

We used interview guide for key informant interviews. We got similar responses by the time we interviewed 12th participants, hence saturation was determined.

We used deductive approach for thematic analysis and followed six-steps (familiarization, coding, generating themes, reviewing themes, defining and naming themes and writing up analysis of data).

The audiotaped interviews were transcribed verbatim independently by two investigators (HKC and SP). The investigators (HKC and SP) reread through the transcripts several times to familiarise themselves with the data. Coding was done by highlighting important phrases or sentences from the transcripts. The independently generated codes were then checked for agreement. We identified the pattern from codes and formulated themes related to women’s empowerment, abortion and family planning knowledge and practices. The themes were further discussed by all investigators and finalized. The findings obtained from two separate analyses were drawn together to form meta inferences.

## Results

Majority (40.2%) belonged to 20–24 years with Mean ± SD of 25.68 ± 6.38 years. Almost 73% were Dalit, 38.3% had informal education and 88.6% were housemaker. Most (67.1%) of them belonged to nuclear family. Almost 95% had ever given birth: 39.3% had ≥ 3 children, 5.1% had stillbirth, 2.6% twin pregnancy and 62% were multiparous. During data collection, 9.2% were pregnant (Table 1).

**Table 1:** Baseline characteristics of participants (n = 316)

Characteristics	n (%)
Age group (year)	Mean ± SD: 25.68 ± 6.38 years
15–19	35 (11.1)
20–24	127 (40.2)
25–29	69 (21.8)
30–34	45 (14.2)
35–39	20 (6.3)
40–44	14 (4.4)
45–49	6 (1.9)
Ethnicity	
Dalit	230 (72.8)
Janajati	42 (13.3)
Muslim	6 (1.9)
Madhesi	38 (12.0)
Education	
Primary	51 (16.1)
Lower secondary	29 (9.2)
Secondary	37 (11.7)
Higher secondary	5 (1.6)
Informal	121 (38.3)
Illiterate	73 (23.1)
Education (head of household)	
Primary	61 (19.3)
Lower secondary	44 (13.9)
Secondary	23 (7.3)
Higher secondary	4 (1.3)
Informal	3 (0.9)
Illiterate	98 (31)
Occupation	
House maker	280 (88.6)
Daily wedge	23 (7.3)
Shop/tailor	8 (2.5)
Factory worker	5 (1.6)
Family type	
Nuclear	212 (67.1)
Joint	104 (32.9)
Occupation (head of household)	
Unskilled worker	138 (43.7)
Semi-skilled worker	116 (36.7)
Skilled worker	45 (14.2)
Clerical, shop-owner, farmer	16 (5.1)
Semi-profession	1 (0.3)
Family income per month (in NPR)	
≤ 2300	1 (0.3)
2301–6850	22 (7.0)
6851–11,450	56 (17.7)
11,451–17,150	114 (36.1)
17,151–22,850	79 (25.0)
22,851–45,750	44 (13.9)
Birth history (Yes)	300 (94.9)
1	87 (29.0)
2	95 (31.7)
≥ 3	118 (39.3)
Still born history (n = 300)	4 (1.3)
Twin pregnancy history (n = 300)	8 (2.6)
Current pregnancy status	29 (9.2)
Parity	
Nulliparous	18 (5.7)
Primiparous	85 (26.9)
Multiparous^a^	196 (62.0)
Grandmultiparous^b^	17 (5.4)

^a^Multiparous: > 1 to 4 times pregnant

^b^Grand multiparous: ≥ 5 times pregnant

We found that 76.9% were literate, 88.6% housemaker, 62.7% upper lower socioeconomic status and 82.6% had early marriage history (12–19 years). With regards to fertility preferences: 91.4% had history of intended pregnancy, 33.1% desire for future pregnancy, 12% had history of under five mortality, 30.1% wish to have two children irrespective of sex of children delivered. In reproductive healthcare decision making: 83.7% couples decide jointly on selecting contraception, 61.1% women were able to refuse sexual intercourse with their husbands, half (50.8%) women could ask their husband to use condoms during intercourse. Most couples (88.9%) had joint decisions on general healthcare seeking behaviours and 90.5% couples jointly decided on using income. More than half (51.6%) did not belong to any social group (Table 2). We found only 27.8% had knowledge on fertile period and 2.5% had knowledge on legal provision of abortion. Almost 14% had history of abortion; among them one participant did not receive post abortion care and family planning services. Seventy-seven percent had knowledge on modern contraceptive methods. Nearly two-third (63.84%) women were currently using contraception, in which majority (41.6%) were Depo-Provera users, 39.3% women had sterilisation and 47.4% were thinking to use it in the near future (Table 3). Current use of modern contraceptives were more among below average women empowerment groups (p 0.041, OR 0.593 C.I. 0.36–0.98). We could not find any statistical significant differences among women’s empowerment with abortion knowledge (p 0.549); family planning knowledge (p 0.495) and women’s decision for future use of modern contraceptives (p 0.977) (Table 4).

**Table 2:** Women empowerment variables

Characteristics	n (%)
Education status (n = 316)	
Illiterate	73 (23.1)
Literate	243 (76.9)
Occupation (n = 316)	
Housemaker	280 (88.6)
Other than housemaker	36 (11.4)
Socio-economic status^c^ (n = 316)	
Upper lower	198 (62.7)
Lower middle	118 (37.3)
Age at marriage (n = 316)	
12–19 years	261 (82.6)
20–30 years	55 (17.4)
Fertility preferences	
Intended pregnancy history(n = 313)^a^	286 (91.4)
Desire for future pregnancy(n = 287)^b^	95 (33.1)
Child mortality history (n = 300)	36 (12.0)
No sex preferences of child (n = 316)	95 (30.1)
Ideal number of children i.e. 2 (n = 316)	95 (30.1)
Reproductive healthcare decision making (n = 312)^d^	
Decision making on using contraception	
Couple	261 (83.7)
Single/In laws	51 (16.3)
Able to refuse undesired sexual intercourse	190 (61.1)
Able to ask husband to use condom during intercourse	158 (50.8)
General healthcare decision making (n = 316)	
Couple	281 (88.9)
Other than couple	35 (11.1)
Decision for income use (n = 316)	
Couple	286 (90.5)
Other than couple	30 (9.5)
Involvement in social group (n = 316)	
Mothers/saving/women’s group	153 (48.4)
Doesn’t belong to any group	163 (51.6)
Women empowerment (n = 316)	
Above average	160 (50.6)
Below average	156 (49.4)

^a^Including at least one previous pregnancy history

^b^Current pregnant status excluded

^c^Modification of Kuppuswamy’s socioeconomic status scale in context to Nepal [16]

^d^Single women and widow excluded

**Table 3:** Knowledge and practice on abortion and family planning

Characteristics	n(%)
Knowledge on fertile period (n = 316)	88 (27.8)
Knowledge on conditions for legal abortion (n = 316)	8 (2.5)
Overall abortion knowledge (n = 316)	92 (29.1)
Post abortion care and family planning services received (n = 43)	42 (97.67)
Knowledge on modern contraceptives (n = 316)	244 (77.2)
Current use of modern contraceptives (n = 271)^a^	173 (63.84)
Methods of family planning used (n = 173)	
Depo-Provera	72 (41.6)
IUCD	4 (2.3)
Norplant	18 (10.4)
OCP	9 (5.2)
Male condom	2 (1.2)
Female sterilization	68 (39.3)
Thinking of using in near future (n = 116)^*b*^	55 (47.4)

^a^Husband going abroad, widow, hysterectomy, and currently pregnant were excluded

^b^Hysterectomy, husband going abroad, widow, current family planning users were excluded

**Table 4:** Association of women empowerment with abortion and family planning

	Abortion knowledge		Post abortion care and family planning counseling received		Family planning knowledge		Current use of modern contraceptives		Thinking for future use of modern contraceptives	
	Yes	No	Yes	No	Yes	No	Yes	No	Yes	No
Women empowerment										
Above average	49 (30.6%)	111 (69.4%)	26 (96.3%)	1 (3.7%)	121 (75.6%)	39 (24.4%)	80 (58%)	58 (42%)	29 (47.5%)	32 (52.5%)
Below average	43 (27.6%)	113 (72.4%)	16 (100%)	0 (0%)	123 (78.8%)	33 (21.2%)	93 (69.9%)	40 (30.1%)	26 (47.3%)	29 (52.7%)
OR (CI)	1.16 (0.713–1.886)		NA^a^		0.832 (0.49–1.41)		0.593 (0.36–0.98)		1.01 (0.49–2.097)	
p value	0.549		NA^a^		0.495		0.041^b^		0.977	

^a^ Not applicable

^b^p value < 0.05 considered statistical significant

### Key informant interviews

Despite quantitative analysis of participants (service utilizers), we were not clear on women’s empowerment perspective from healthcare providers and community leaders. The community leaders have a major role in policy level decision-making and health care providers at service delivery. We conducted key informant interviews to explore further the women’s current issues, factors that may contribute to empowerment of women, their reproductive health status mainly in part of abortion and family planning, the available reproductive health facilities and its utilisation by marginalized women of that locality. The themes identified from key informant interviews further clarified status of women’s empowerment and explore abortion and family planning knowledge and practice.

### Characteristics of participants

Key informant interviews were conducted with 15 key persons of the same community where eight were healthcare providers and seven were local leaders. The mean age of participants was 44.67 years with standard deviation of 13.38 years ranging from 26 to 62 years. Majority of them were male (60%) and all were literate.

### Present status of marginalized women

Majority of the informants said illiteracy, poverty and domestic violence are the major issues of marginalized women. They are completely dependent on their husband and had to tolerate any types of physical or emotional violence. The quotations highlight the reasons behind lack of women empowerment. *The status of women specially marginalized women in our society is very poor. The main reason is lack of education and poverty. Our ward is under municipality but the status of the society is worse than the village development committee (VDC) because the population and the area coverage of our ward is bigger and we have access to only one health post here. (Local leader, Male).*

*The present status of marginalized women is poor day by day, Ammm…. Loan support from nongovernmental organisations (NGOs), international nongovernmental organisations (INGOs), co-operative limited did somehow help in earlier days but recently all the families are being affected by the poor socioeconomic status and inability to pay loans offered by the banks. (Local leader, Male)*

### Abortion knowledge and practice

One informant reported, abortion is common among 20–35 years female in their locality. Sometimes abortion is practiced illegally after sex determination even at 3–4 months of pregnancy. One health worker often stated that unmarried teenagers, factory workers even seek to health posts with complication of induced illegal abortion. Causes of abortion was unwanted pregnancy, unmet family planning and default of depo provera. As stated by healthcare providers, DPHO, IPAS has been constantly supporting abortion services in some primary health centers (PHC) and they often do have medical abortion and urinary pregnancy test services available. The quotations highlight the reason and sequelae of abortion.

*“Abortion is practiced with some native techniques or with use of herbals at home by some females and come to health centers with complication like bleeding, infection or incomplete expulsion.” (Healthcare Provider, Female)*

*“Nowadays extra marital sexual practices have landed up with many abortion cases.”(Healthcare Provider, Male)*

*Many attempt to take medicines at home from nearby clinics to avoid disclosure. While many did not visit local health centers due to stigma and fear of unacceptance in society. (Healthcare Provider, Female)*

*“Some practiced abortion after sex determination illegally.” (Healthcare Provider, Female)*

One of the Muslim local leader stated that their society do not consider abortion as a good practice even though the people have some knowledge in it.

*We do not consider abortion as good things in our society. If anyone had induced abortion in 2 or 3 months of pregnancy, our society views them as a social stigma. However, people are a little aware and are positive regarding abortion nowadays, but abortion practice is uncommon in our society. (Local leader, Male)*

### Family planning knowledge and practice

Many informants said that people’s awareness on family planning methods is improving. The common methods available were copper T, male condom, injection (Depo Provera), one is electric operation (Tubectomy) and one is hand operation (Vasectomy). These services has been provided by PHCs, FCHVs, mobile camps, NGOs, INGOs, Marie Stopes and Zonal hospitals. However, people demand for all types of free of cost family planning services in their nearby primary healthcare centers. The quotations highlight the commonly used family planning devices, its availability and preferences.

*We have copper-T, male condom, injection, one is electric operation and one is hand operation. Some are using these methods and some not. We still have a lack of public awareness on these methods. Most (80%) seek services from primary healthcare centers and 20% goes to Koshi Zonal Hospital. (Local leader, Female)*

*“People demand for free of cost availability of modern family planning services in health centers” (Healthcare Provider, Male)*

*“Mobile camp on permanent sterilization is organised sometimes in community health centers but very often they have to take service from Marie Stopes.” (Healthcare Provider, Female)*

Cafeteria choice for family planning was not into practice. Female users were more than male users. Many couples practiced natural methods like lactational amenorrhea, withdrawal and emergency contraception. They preferred to take temporary methods, especially depo-provera.

*Females use more family planning methods than male. (Local leader, Male)*

*“Many people use family planning only after 2–3 children.” (Healthcare Provider, Female Community Health Volunteer)*

*“The client's mind set is already fixed on using Depo-Provera, they don’t want to hear about any other methods.” (Healthcare Provider, Male)*

*“Even elderly females prefer using depo knowingly and unknowingly.” (Healthcare Provider, Female Community Health Volunteer)*

*“Yes, many newly married male come to me and ask for advice on natural methods. They were not satisfied with family planning devices thinking of its side effects and decrease in sexual pleasure.” (Healthcare Provider, Female)*

*Most clients asked us—Why don’t you have provision of distributing emergency contraception pills in your health center? We demand for these services.” (Healthcare Provider, Male)*

## Discussion

Nepal is continuously striving to improve women’s status within the society; there remains a disparity and inequality among disadvantaged and marginalized groups. It is very essential to cover all spectrums of women in order to improve women’s status within the country. We measured women’s empowerment in the marginalized communities of Morang district of Nepal. Our study found that women’s empowerment above vs below median was almost the same (50.6% vs 49.4% respectively). Our key informant interviews further suggested that poverty, illiteracy, unemployment and dependence on husbands were common issues among marginalized women. Improvement in women’s education will give higher opportunities, increase access to financial resources and autonomous decision making over their life issues. Employment and having an income are important contributors to improve maternal and child health. Despite variation in defining women’s empowerment; evidence suggests that there is a two way relationship between women’s empowerment and fertility i.e. lower fertility leads to higher women’s empowerment and vice versa [17, 18]. Fertility choice is linked with reduced rate of unintended pregnancy, unsafe abortion and women’s decision on use of family planning devices. Women’s autonomy in decision making over consensual sexual relations, contraception use and access to sexual and reproductive health services is key to their empowerment and the full exercise of their reproductive rights [19]. A woman’s ability to say “no” to her husband/partner if she does not want to have sexual intercourse, decisions being made “mainly by the partner”, as opposed to decision being made “by the husband or wife alone" on contraceptives use is well aligned with the concept of sexual autonomy and women’s empowerment [19]. In this study, more than four-fifth couples decided jointly on using contraception, three-fifth women were able to refuse sexual intercourse with their husbands. Women who make their own decision regarding healthcare seeking for themselves are considered empowered to exercise their reproductive rights [19].

We found no statistical significant difference between women’s empowerment and knowledge on abortion. Knowledge on abortion law is less among marginalized women, which may affect their abortion practices. Our key informants reported illegal abortion among teenagers and factory workers was common which was further confirmed by their visit at health post and primary health centers for post abortion services for its complications. Some of our study areas have abundant factories where female workers are more vulnerable for illegal pregnancy due to unsafe sexual practice because of peer pressure, as a source of income, extramarital affairs etc. People also hesitate to utilize abortion services due to deep-rooted social stigma prevailed in these communities. Further analysis of Nepal demography and health survey reported that 40% of women aged 15–49 years had knowledge on abortion law compared to our finding (2.5%). It is because our study participants belonged to marginalized communities where women’s empowerment is not adequate [20]. Even after the legislation of abortion in Nepal since 2002, unsafe abortion remains the third highest (7%) direct cause of maternal death in Nepal [21]. Similar type of finding reported from our key informants where many such incidence goes unnoticed and unreported. Different studies conducted in Nepal suggested that patriarchal system, low literacy rates, social and cultural norms, limited sexual reproductive health and rights, lack of women’s autonomy and knowledge, poor socio-economic status, geographic isolation and stigma were the common reason for unsafe abortion practice [22–24].

We found more than three fourth had knowledge on modern family planning methods which is similar to national data [5]. National survey found more than three fourth of reproductive age women intended future use of family planning whereas we found only about half. Our study found that more than 60% were the current users of modern contraceptives where Depo Provera and female sterilisation were most common. National survey reported 43% modern contraceptives users where most commonly used were female sterilisation and Depo Provera [5]. The use of Depo Provera in our study is more which may be due to easy availability at nearby pharmacies. The lack of practice of cafeteria choice limits the selection of contraceptive methods as reported by our key informant form health care providers group. In this study, increment in female sterilization is due to the mobile camps organised by various government and non-government organisations, practice of early age marriage and influence from their society. Studies reported that caste based discrimination and continuing social exclusion resulted no visit to health facilities by ethnical minorities to avoid potential discrimination and poor quality care [25–27]. A study from Nepal suggested that women currently not working, poor, Muslim and Janajati ethnicity, and lack of autonomy in household decision-making were not using family planning methods [28]. Various studies from other places reported that women factors like higher education, good socioeconomic status, involvement in household decision making, autonomy on their health seeking behaviour, participation in fertility choices, participation in income generating activities, access to information and country’s socio-cultural and health system context are more likely to decide and use contraceptive methods [29–31]. We did not find any significant difference between women’s empowerment with knowledge and practice of family planning methods. It is supported by a demography and health survey (DHS) report of Sub Saharan Africa where women’s empowerment was less important in determining use of family planning; attaining higher education was not much important in choosing effectiveness of family planning methods and country-specific norms may restrict women in their decision-making capacities [31]. In contrast to our study, increased use of family planning methods was found with increased education and higher economic status [26]. In our study, key informant interviews further explored the possible explanation for no difference in the use of family planning methods between below median women’s empowerment group and above median empowerment group which might be due to societal influences, geographical feasibility of family planning services, easy transportation, prime focused of various governmental and non governmental organization on reproductive health programmes, nearby health centers, mobile camps and practicing sterilization because of their early age reproduction and fulfilment of desired children.

### Strength of the study

Assessment of women’s empowerment at marginalized communities is challenging. To the best of our knowledge, this is for the first time in the communities of eastern Nepal conducting research among marginalized women incorporating fifteen variables that are related to women’s empowerment together. We did mixed method approach, because the certain issues of women’s empowerment, knowledge and practice of family planning and abortion can be less expressed by the study participants. We verified these issues and asked for in-depth study through key informant interviews of relevant stakeholders. This approach strengthens our research findings.

### Limitation of the study

We failed to include husband perspectives because family planning and abortion is couple decision. Due to the unavailability of recorded population of marginalized groups in Morang district stratification with proportionately allocation of the sample could not be done.

### Future research suggestions

We can conduct case control study to find the association of factors with women’s empowerment in marginalized communities. The relationship and interaction between those factors will help to find the in-depth knowledge of enabling and disabling factors of women’s empowerment. We need to search for how the different organisation improve the utilization of family planning and abortion care services in these communities. The role of husband or family member for women’s empowerment needs to be explored. The impact of easy availability of family planning and abortion services at nearby communities for their utilization need to be explored. Similar types of study can be conducted in a larger context in different settings. We recommend systematic review and meta-analysis of research related to marginalized communities of Nepal and abroad to pinpoint the findings.

## Conclusions

More than half women were empowered in our study. Women’s empowerment have no significant role for abortion and family planning among marginalized women. Our key informants further revealed that illiteracy, poverty and domestic violence were contributory factors for hindrance to women’s empowerment. It further explored that marginalized women have poor knowledge on abortion and there is deep rooted social stigma and religious barriers on abortion practice. Illegal abortion was being practiced by unmarried teenagers and factory workers. Irrespective of whether they are empowered or not, nearby facilities of government and non-government family planning services have made easy accessibility and utilization of family planning services among the marginalized women.

### Recommendations

Encouragement, social inclusion, women’s education, capacity building training and job opportunities should be provided to empower women of marginalized communities. The concerned authorities and health service providers should organize health education and awareness programs on abortion, family planning and other women’s reproductive empowerment issues. Equity based health care awareness programs and health care services in all strata of community at local and national level is important to fulfill the gap between marginalized and non-marginalized communities.

## Data Availability

The datasets used and/or analysed during the current study are available from the corresponding author on reasonable request.

## Abbreviations

SDG: Sustainable Development Goal
ICPD: International Conference on Population and Development
MDG: Millennium Development Goal
NDHS: Nepal Demographic and Health Survey
IPV: Intimate partner violence
DPHO: District Public Health Office
FCHV: Female Community Health Volunteers
COREQ: Consolidated Criteria for Reporting Qualitative Research
IUCD: Intrauterine contraceptive devices
OCP: Oral contraceptive pills
VDC: Village development committee
NGO: Nongovernmental organisation
INGO: International nongovernmental organisation
PHC: Primary health center
DHS: Demographic and Health Survey

## References

[1] Tshuma D, Kajala E. Empower women—five major components of women’s empowerment. EmpowerWomen. https://www.empowerwomen.org/en/community/discussions/2016/11/five-major-components-of-womens-empowerment. Accessed 14 Apr 2020.

[2] Alsop R, Heinsohn N. Measuring empowerment in practice: structuring analysis and framing indicators. Policy Research Working Papers. 2005. 10.1596/1813-9450-3510.

[3] International Conference on Population and Development (ICPD. United Nations Population Fund. 1994. https://www.unfpa.org/events/international-conference-population-and-development-icpd. Accessed 12 Apr 2020.

[4] Martin BV. United Nations: gender equality and women’s empowerment. United Nations Sustainable Development. https://www.un.org/sustainabledevelopment/gender-equality/. Accessed 17 Apr 2020.

[5] Ministry of Health, Nepal, New ERA and ICF. Nepal Demographic and Health Survey. Kathmandu, Nepal: Ministry of Health, Nepal; 2017. https://www.dhsprogram.com/pubs/pdf/fr336/fr336.pdf. Accessed 14 Apr 2020.

[6] Government of Nepal National Planning Commission Secretariat Central Bureau of Statistics Ramshah Path, Kathmandu, Nepal. Population Monograph of Nepal Volume 1 Population dynamics. 2014. https://nepal.unfpa.org/sites/default/files/pub-pdf/PopulationMonograph2014Volume1.pdf. Accessed 2 Apr 2020.

[7] Dalit Welfare Organization. Dalit Welfare Organisation—NGO, Nepal—eliminating caste discrimination. Dalit Welfare Organization (DWO). http://dwo.org.np/v1/dalit.php. Accessed 14 Apr 2020.

[8] Ministry of Health, Government of Nepal. Nepal Health Sector Strategy Plan 2016–2021. Ministry of Health, Government of Nepal; 2017. http://www.nhssp.org.np/NHSSP_Archives/health_policy/NHSS_implementation_plan_2016_2021_february2017.pdf. Accessed 13 Apr 2020.

[9] Fisher B, Naidoo R (2016). The geography of gender inequality. PLoS ONE.

[10] Derek Osborn Amy Cutter. Universal sustainable development goals understanding the transformational challenge for developed countries. 2015. https://sustainabledevelopment.un.org/content/documents/1684SF_-_SDG_Universality_Report_-_May_2015.pdf. Accessed 1 Apr 2020.

[11] Biswas TK, Kabir M (2002). Women’s empowerment and current use of contraception in Bangladesh. Asia-Pac J Rural Dev.

[12] Hameed W, Azmat SK, Ali M, Sheikh MI, Abbas G, Temmerman M (2014). Women’s empowerment and contraceptive use: the role of independent versus couples' decision-making, from a lower middle income country perspective. PLoS ONE.

[13] Blanc AK (2001). The effect of power in sexual relationships on sexual and reproductive health: an examination of the evidence. Stud Fam Plann.

[14] Tong A, Sainsbury P, Craig J (2007). Consolidated criteria for reporting qualitative research (COREQ): a 32-item checklist for interviews and focus groups. Int J Qual Health Care.

[15] Sebayang, Susy K., Ferry Efendi, and Erni Astutik. Women’s Empowerment and the Use of Antenatal Care Services in Southeast Asian Countries. Rockville, Maryland, USA: ICF; 2017. Report No.: DHS Working Paper No. 129. https://www.dhsprogram.com/pubs/pdf/WP129/WP129.pdf. Accessed 12 Apr 2020.

[16] Tulika S, Sharma S (2017). Socio-economic status scales updated for 2017. Int J Res Med Sci.

[17] Atake E-H, Ali PG (2019). Women’s empowerment and fertility preferences in high fertility countries in Sub-Saharan Africa. BMC Womens Health..

[18] Phan LD. Women’s empowerment and fertility preferences in Southeast Asia. 2016. http://hdl.handle.net/2123/15251. Accessed 11 Apr 2020.

[19] The Global Health Observatory. World Health organisation. https://www.who.int/data/gho/indicator-metadata-registry/imr-details/4986. Accessed 11 Apr 2020.

[20] Pandey JP, Dhakal MR, Karki S, Poudel P, Pradhan MS. Maternal and Child Health in Nepal: the effects of caste, ethnicity, and regional identity further analysis of the 2011 Nepal Demographic and Health Survey. Kathmandu, Nepal March 2013. https://www.dhsprogram.com/pubs/pdf/FA73/FA73.pdf. Accessed 7 Apr 2020.

[21] Pradhan A, Suvedi BK, Barnett S, Sharma SK, Puri M, Poudel P, Shovana Rai Chitrakar Naresh Pratap KC, Hulton L. Nepal maternal mortality and morbidity study 2008/2009. 2010. http://nnfsp.gov.np/PublicationFiles/aaef7977-9196-44d5-b173-14bb1cce4683.pdf. Accessed 11 Apr 2020.

[22] Thapa S, Sharma SK, Khatiwada N (2014). Women’s knowledge of abortion law and availability of services in Nepal. J Biosoc Sci..

[23] Puri M, Regmi S, Tamang A, Shrestha P (2014). Road map to scaling-up: translating operations research study’s results into actions for expanding medical abortion services in rural health facilities in Nepal. Health Res Policy Syst..

[24] Thomas D, Bell S, Dahal K, Grellier R, Jha C, Prasai S, Subedi HN. Voices from the community: access to health services a rapid participatory ethnographic evaluation and research (PEER) study, Nepal. Government of Nepal (GoN), Population Division Ministry of Health and Population (MoHP), with support from Nepal Health Sector Support Programme (NHSSP); 2012. http://www.nhssp.org.np/NHSSP_Archives/gesi/PEER_study_2012.pdf. Accessed 11 Apr 2020.

[25] Sharma SK, Ghimire DR, Pratap N (2011). Ethnic differentials of the impact of Family Planning Program on contraceptive use in Nepal. Demogr Res..

[26] Najafi-Sharjabad F, Zainiyah Syed Yahya S, Abdul Rahman H, Hanafiah Juni M, Abdul Manaf R (2013). Barriers of modern contraceptive practices among Asian women: a mini literature review. Glob J Health Sci..

[27] Adhikari R, Acharya D, Lal Ranabhat C, Ranju KC. Factors associated with non-use of contraceptives among married women in Nepal. Journal of Health Promotion. 2019;7. https://www.nepjol.info/index.php/jhp/article/view/254. Accessed 12 Apr 2020.

[28] Corroon M, Speizer IS, Fotso J-C, Akiode A, Saad A, Calhoun L (2014). The role of gender empowerment on reproductive health outcomes in urban Nigeria. Matern Child Health J..

[29] Yaya S, Uthman OA, Ekholuenetale M, Bishwajit G (2018). Women empowerment as an enabling factor of contraceptive use in sub-Saharan Africa: a multilevel analysis of cross-sectional surveys of 32 countries. Reprod Health..

[30] Pratley P (2016). Associations between quantitative measures of women’s empowerment and access to care and health status for mothers and their children: a systematic review of evidence from the developing world. Soc Sci Med..

[31] Larsson C, Stanfors M (2014). Women’s education, empowerment, and contraceptive use in sub-Saharan Africa: findings from recent demographic and health surveys. Etude Popul Afr..

